# Transient Electrocardiographic Changes in Anaphylaxis Mimicking Acute Coronary Syndrome: A Case Series

**DOI:** 10.5811/cpcem.61871

**Published:** 2026-10-13

**Authors:** S Hari Ganesh, V T Amrithanand, B G Kowsthubha

**Affiliations:** Jawaharlal Institute of Postgraduate Medical Education and Research, Department of Emergency Medicine and Trauma, Puducherry, India

**Keywords:** anaphylaxis, electrocardiography, acute coronary syndrome mimic, epinephrine, Kounis syndrome

## Abstract

**Introduction:**

Anaphylaxis is a life-threatening emergency that can involve significant cardiovascular manifestations. Transient electrocardiographic (ECG) changes during anaphylaxis and following epinephrine administration may closely mimic acute coronary syndrome, creating a diagnostic dilemma in the emergency department (ED). Failure to recognize these reversible patterns can result in delayed anaphylaxis treatment or unnecessary activation of invasive cardiac pathways.

**Case Series:**

We report a case series of three adult patients presenting to the ED with anaphylaxis who developed alarming ECG changes, including ST-segment elevation, ST-segment depression, and hyperacute T waves. One patient demonstrated transient ECG changes temporally related to intramuscular epinephrine administration, while two patients exhibited features suggestive of hypersensitivity-associated coronary vasospasm or thrombosis manifesting as ECG changes. In all cases, serial ECGs, high-sensitivity cardiac troponin testing, and focused echocardiography revealed resolution of ECG abnormalities without dynamic biomarker elevation or regional wall motion abnormalities. All patients improved with prompt anaphylaxis-directed therapy and were managed conservatively without coronary intervention.

**Conclusion:**

Transient ECG changes during anaphylaxis and after epinephrine administration can closely resemble acute coronary syndrome but may resolve completely with appropriate allergy-directed treatment. Serial ECG assessment, cardiac biomarkers, and focused echocardiography are essential tools for emergency physicians to differentiate transient allergic or catecholamine-mediated effects from true myocardial ischemia. Recognition of these patterns allows timely treatment of anaphylaxis while avoiding unnecessary invasive cardiac interventions.

## INTRODUCTION

Anaphylaxis is a severe, life-threatening hypersensitivity reaction characterized by rapid involvement of multiple organ systems, most notably the respiratory and cardiovascular systems. Cardiovascular manifestations are a major cause of morbidity and mortality in anaphylaxis and include hypotension, distributive shock, and cardiac dysfunction resulting from the systemic release of inflammatory mediators. Experimental and clinical evidence suggests that the heart, particularly the coronary circulation, functions as a primary target organ during anaphylaxis, making cardiac involvement both common and clinically significant.^1^

Electrocardiographic (ECG) abnormalities during anaphylaxis are increasingly recognized but remain underreported in routine clinical practice. These changes may include ST-segment elevation or depression, hyperacute T waves, arrhythmias, and conduction disturbances.^2^ Such findings can closely resemble acute coronary syndrome (ACS), especially when accompanied by chest pain, diaphoresis, hypotension, or syncope. The overlap between anaphylaxis and ACS presentations creates a diagnostic challenge in the emergency department (ED), where rapid decision-making is essential and misclassification may lead to either harmful delays in anaphylaxis treatment or unnecessary invasive cardiac interventions.^3^

Multiple mechanisms have been proposed to explain ECG changes in anaphylaxis, including coronary hypoperfusion due to systemic vasodilation and plasma extravasation, direct myocardial depression mediated by inflammatory cytokines, coronary vasospasm, and platelet activation.^4^ In some patients, these processes manifest as Kounis syndrome, an allergic ACS characterized by coronary vasospasm, plaque rupture, or stent thrombosis triggered by hypersensitivity reactions.^5^–^7^ Epinephrine, while life-saving in anaphylaxis, exerts complex cardiovascular effects through its α- and β-adrenergic receptor activity. Stimulation of β1-adrenergic receptors increases heart rate, myocardial contractility, and conduction velocity, collectively raising cardiac workload and myocardial oxygen demand. Simultaneously, α-adrenergic-mediated coronary vasoconstriction reduces myocardial oxygen supply, creating a supply-demand mismatch that may precipitate transient subendocardial ischemia, particularly in susceptible individuals. Additional prothrombotic effects, including enhanced platelet aggregation and thromboxane B2 production, may further compromise coronary microcirculatory flow. Together, these mechanisms can produce transient ECG changes—ranging from ST-segment depression and T wave abnormalities to arrhythmias—that may mimic ACS but typically resolve with hemodynamic stabilization and do not reflect irreversible myocardial injury.^8^–^10^

More recently, these overlapping entities have been conceptualized as part of the ATAK complex—adrenaline, takotsubo cardiomyopathy, anaphylaxis, and Kounis syndrome—highlighting the intricate interplay between allergic reactions, catecholamine exposure, and cardiac dysfunction.^11^ Distinguishing among these mechanisms in the acute setting remains challenging, particularly because many anaphylaxis-related ECG changes are transient and resolve with appropriate treatment, often in the absence of significant troponin elevation or echocardiographic abnormalities.

Given the potential for diagnostic confusion and management error, recognition of transient ECG changes associated with anaphylaxis and its treatment is critical for emergency physicians. We present a case series of three patients with anaphylaxis who developed reversible ECG changes during their ED course, illustrating key clinical features that aid differentiation from true ACS and emphasizing the importance of continuous cardiac monitoring and temporal ECG assessment during anaphylaxis management.

*CPC-EM Capsule*

What do we already know about this clinical entity?

*Anaphylaxis can rarely cause transient echocardiogram (ECG) abnormalities and mimic acute coronary syndrome (ACS) through allergic cardiac involvement.*

What makes this presentation of disease reportable?

*This case series demonstrates dynamic ECG changes during anaphylaxis that closely resembled ACS but resolved after treatment.*

What is the major learning point?

*Transient ECG abnormalities during anaphylaxis should be interpreted with clinical context before initiating ACS-specific interventions.*

How might this improve emergency medicine practice?

*Early recognition of anaphylaxis-related ECG changes may prevent misdiagnosis and ensure prompt adrenaline-centered treatment.*

## CASE SERIES

### Case 1: Epinephrine-Related Transient ECG Changes

ED Presentation: A 62-year-old man with no known prior comorbidities presented to the ED with sudden onset generalized pruritus, urticarial wheals, profuse diaphoresis, and left periorbital edema shortly after food intake and ingestion of oral analgesics earlier in the day. He had no chest pain. On arrival, he was diaphoretic and dyspneic, unable to complete sentences, with a weak pulse and unrecordable blood pressure, and oxygen saturation consistent with anaphylactic shock involving mucocutaneous, respiratory and cardiovascular systems. Physical examination was notable for left periorbital edema; cardiovascular, respiratory, and abdominal examinations were otherwise unremarkable. The initial 12-lead ECG demonstrated normal sinus rhythm at 72 beats per minute with a normal axis and no acute ST–T wave abnormalities (Image 1A). Presumed anaphylaxis was treated immediately with intramuscular epinephrine 0.5 mg (1:1,000) administered into the anterolateral thigh, along with intravenous (IV) crystalloids, IV chlorpheniramine 10 mg, and IV hydrocortisone 200 mg with continuous cardiac monitoring was initiated.

Shortly after epinephrine administration, cardiac monitoring demonstrated new ischemic changes. A repeat ECG showed sinus bradycardia at 52 beats per minute with right axis deviation and diffuse ST-segment depression in leads V1–V6, without posterior or right ventricular lead involvement (Image 1B). As blood pressure improved, a subsequent ECG obtained 30 minutes later demonstrated resolution of ST changes with sinus rhythm at 66 beats per minute and an RSR′ pattern in V1–V2 (Image 1C).

High-sensitivity troponin I was borderline elevated at 19.98 ng/L (reference range, <13 ng/L) without dynamic rise on repeat testing. Transthoracic echocardiography revealed normal left ventricular systolic function with no regional wall motion abnormalities.

After cardiology consultation, the patient was managed conservatively without antiplatelet therapy, anticoagulation, or coronary intervention. Electrocardiogram changes and symptoms resolved completely, and the findings were attributed to transient epinephrine-related myocardial effect in the setting of anaphylaxis.

### Case 2: Transient ECG Changes Consistent with Kounis Syndrome

A 34-year-old man with no known prior cardiac history presented to the ED with acute-onset left-sided chest pain, generalized pruritus, and diffuse erythematous rash. Symptoms developed within hours of re-exposure to oral amoxicillin–clavulanate prescribed for dental pain. Chest pain was associated with diaphoresis, nausea, and vomiting. On arrival, he was hemodynamically stable with a heart rate of 100 beats per minute, blood pressure of 110/60 mm Hg, and oxygen saturation of 98% on room air. Physical examination revealed a diffuse erythematous rash; chest auscultation was clear with no added sounds, cardiac auscultation was normal, and the abdomen was soft and nontender. The presenting 12-lead ECG demonstrated sinus tachycardia at 100 beats per minute with ST-segment elevation in leads II, III, aVF, and V1–V6, ST depression in leads I and aVL, and hyperacute T waves, including right-sided precordial leads (Image 2A). Given clinical features consistent with anaphylaxis and concurrent ischemic-appearing ECG changes, the patient was treated with intramuscular epinephrine 0.5 mg, IV chlorpheniramine 10 mg, IV hydrocortisone 200 mg, and continuous cardiac monitoring was initiated.

Following clinical stabilization, a repeat ECG demonstrated normalization of ST-segment and T wave morphology (Image 2B). High-sensitivity troponin I measured 7 ng/L, with no subsequent rise on serial testing. Transthoracic echocardiography showed normal biventricular function without regional wall motion abnormalities. In consultation with cardiology, conservative management was continued, as ECG changes were transient and biomarkers remained negative. The presentation was considered consistent with type I Kounis syndrome, with resolution following treatment of the allergic reaction.

### Case 3: Anaphylaxis-Associated Transient Ischemic ECG Changes

A 40-year-old man with a recent history of insect bite was brought to the ED with profuse diaphoresis and generalized pruritis without associated chest pain. On arrival, patient was hemodynamically unstable with an unrecordable blood pressure and a heart rate of 109 beats per minute and an unrecordable oxygen saturation in the setting of severe hypotension, consistent with anaphylactic shock involving the cutaneous and cardiovascular systems. Physical examination was notable for generalized urticaria; respiratory and abdominal examinations were unremarkable. The initial 12 lead ECG demonstrated diffuse ST-segment depression with hyperacute T waves in leads V2–V3 (Image 3A).

Immediate resuscitation was initiated with intramuscular epinephrine 0.5 mg (1:1,000) administered to the anterolateral thigh, along with IV fluids bolus of 500 mL followed by maintenance infusion at 150 mL/hour, IV chlorpheniramine 10 mg, and IV hydrocortisone 200 mg, with continuous ECG monitoring maintained throughout. A repeat ECG obtained approximately 30 minutes after clinical stabilization demonstrated complete resolution of hyperacute T waves and ST-segment changes (Image 3B).

Cardiac biomarkers were not elevated, and bedside echocardiography revealed preserved biventricular function without regional wall motion abnormalities. Cardiology was consulted, and in the absence of chest pain, dynamic biomarker rise, or echocardiographic abnormality, conservative management was recommended. The patient’s hemodynamics stabilized with resolution of symptoms and ECG abnormalities. He was discharged after observation for six hours with a diagnosis of transient anaphylaxis-related ECG changes, without evidence of ACS.

## DISCUSSION

Electrocardiographic abnormalities during anaphylaxis are increasingly recognized yet remain diagnostically challenging in the ED because they can closely mimic ACS. These changes are often transient and may arise from multiple overlapping mechanisms, including systemic vasodilation with coronary hypoperfusion, direct myocardial depression mediated by inflammatory mediators, coronary vasospasm, and the effects of exogenously administered epinephrine.^1^,^5^ In the acute setting, distinguishing among these mechanisms is critical, as misinterpretation may result in delayed treatment of anaphylaxis or unnecessary activation of invasive cardiac pathways.

In anaphylaxis, cardiovascular collapse is primarily driven by systemic vasodilation, increased vascular permeability, and intravascular volume depletion, leading to reduced preload and impaired coronary perfusion. Experimental and clinical data support the concept that the heart—particularly the coronary circulation—is a direct target of anaphylactic mediators, resulting in global myocardial dysfunction rather than focal ischemia.^1^,^12^ This pathophysiology explains why many patients exhibit transient ischemic-appearing ECG changes with normal or minimally elevated cardiac biomarkers and absence of regional wall motion abnormalities on echocardiography, as observed in our cases.

Kounis syndrome represents a distinct but related entity in which allergic mediator release precipitates coronary vasospasm, plaque rupture, or stent thrombosis, leading to true myocardial ischemia or infarction.^5^–^7^ In contrast to transient anaphylaxis-related ECG changes, Kounis syndrome is more likely to demonstrate persistent ECG abnormalities, dynamic troponin elevation, and regional wall motion abnormalities, reflecting myocardial injury. In our series, only one patient presented with chest pain and ECG findings consistent with Type I Kounis syndrome, yet even in this case, ECG changes resolved rapidly and cardiac biomarkers remained within normal limits, supporting conservative management.

Epinephrine, the cornerstone of anaphylaxis treatment, introduces additional diagnostic complexity. Through its α- and β-adrenergic effects, epinephrine can induce coronary vasoconstriction, increase myocardial oxygen demand, and promote platelet aggregation, resulting in transient ischemic ECG changes or, rarely, myocardial infarction.^8^–^10^ These effects are typically short-lived when epinephrine is administered intramuscularly at recommended doses, as demonstrated in Case 1, where ECG changes appeared shortly after administration and resolved as the pharmacologic effect diminished. Recognition of this temporal relationship is essential to avoid inappropriate escalation to conventional ACS pathways.

From an ED perspective, this case series highlights the importance of serial assessment rather than isolated ECG interpretation. In selected patients with anaphylaxis who demonstrate transient ECG changes, a combination of serial ECGs, high-sensitivity troponin measurements, and focused echocardiography may be sufficient to exclude ongoing myocardial ischemia. Resolution of ECG abnormalities, absence of dynamic troponin rise, and normal ventricular function strongly favor transient anaphylaxis- or epinephrine-related effects over ACS. This approach allows emergency physicians to prioritize life-saving anaphylaxis treatment while safely deferring invasive cardiac evaluation.

Importantly, not all patients with ECG changes during anaphylaxis are appropriate for conservative management. Immediate cardiology involvement and consideration of catheterization are warranted when ECG changes persist despite resolution of anaphylaxis, when there is ongoing chest pain, dynamic elevation of cardiac biomarkers, new regional wall motion abnormalities, or hemodynamic instability unexplained by distributive shock. In such cases, Kounis syndrome or concurrent ACS must be strongly considered.^5^,^7^,^13^

These overlapping entities—anaphylaxis, epinephrine-induced myocardial effects, Kounis syndrome, and stress-induced cardiomyopathy—have been conceptualized as the ATAK complex, underscoring the interconnected nature of allergic and catecholamine-mediated cardiac pathology.^11^ Awareness of this spectrum is essential for emergency physicians, as management decisions must be individualized and guided by clinical trajectory rather than a single diagnostic test. The flowchart on approach is summarized in the figure.

## CONCLUSION

Anaphylaxis and its treatment with intramuscular epinephrine can produce transient electrocardiographic changes that closely mimic acute coronary syndrome, creating a high-risk diagnostic scenario in the emergency department. Recognition of the temporal resolution of ECG abnormalities, in conjunction with serial electrocardiography, high-sensitivity troponin testing, and focused echocardiography, allows emergency physicians to safely distinguish transient anaphylaxis- or catecholamine-mediated effects from true myocardial ischemia in selected patients. Importantly, avoidance of reflexive activation of invasive cardiac pathways should not delay definitive anaphylaxis treatment, while early cardiology involvement remains essential when ECG changes persist, biomarkers rise dynamically, or regional wall motion abnormalities are present, raising concern for Kounis syndrome or concurrent acute coronary syndrome. Through vigilant monitoring and trajectory-based decision-making, emergency physicians can prevent both undertreatment of anaphylaxis and unnecessary cardiac interventions.

## Figures and Tables

**Figure.**
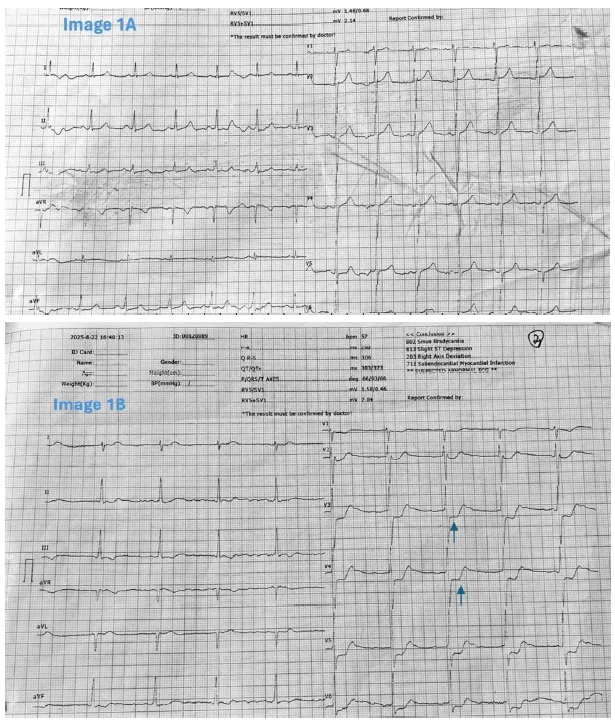
Emergency department flowchart: approach to anaphylaxis with abnormal ECG. *ACS*, acute coronary syndrome; *ECG*, electrocardiogram; *ECHO*, echocardiography; *ED*, emergency department; *hs*, high sensitivity; *IM*, intramuscular; *IV*, intravenous; *min*, minutes; *RWMA*, regional wall motion abnormality.

**Image 1:**
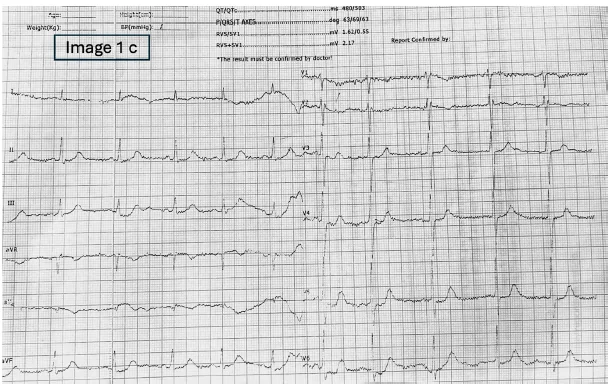
A) The initial 12-lead electrocardiogram (ECG) demonstrated normal sinus rhythm at 72 beats per minute, normal axis, and no acute ST-segment or T wave abnormalities. B) A repeat ECG showed sinus bradycardia at 52 beats per minute with right axis deviation and diffuse ST-segment depression in leads V1–V6. Blue arrow pointing ST-segment depression in lead V3 and V4.

**Image 1C:**
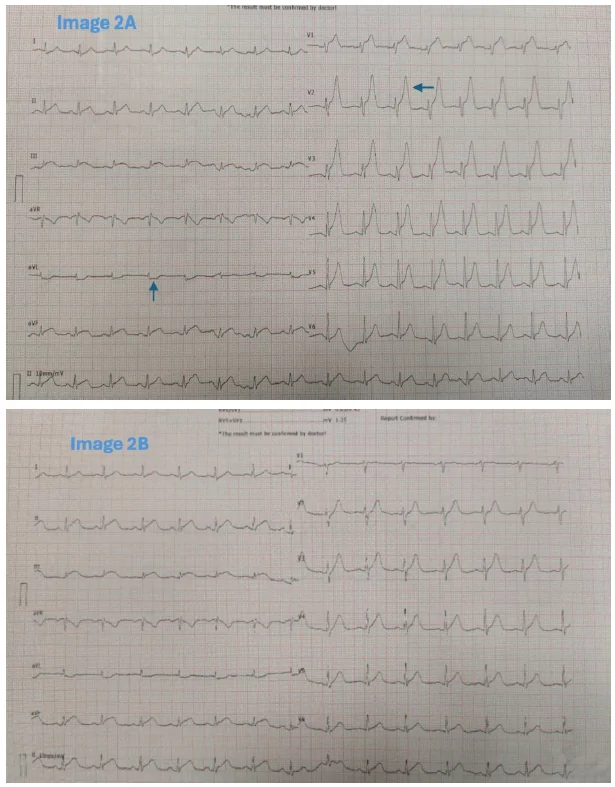
Repeat 12-lead electrocardiogram obtained 30 minutes after epinephrine administration demonstrating resolution of the previously noted ST-segment depression, with restoration of sinus rhythm at 66 beats per minute.

**Image 2:**
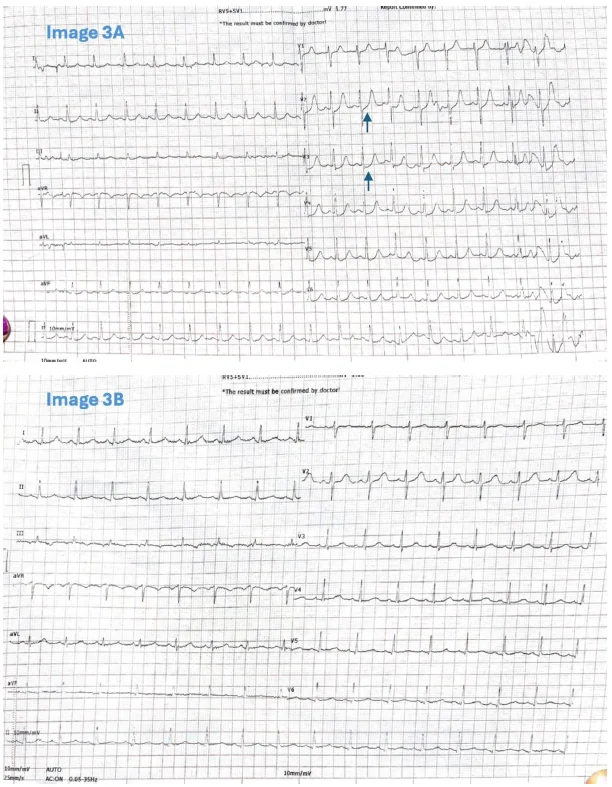
A) The presenting 12-lead electrocardiogram demonstrated sinus tachycardia at 100 beats per minute with ST-segment elevation in leads II, III, aVF, and V1–V6, ST-segment depression in leads I and aVL, and hyperacute T waves, including right-sided precordial leads. Blue arrow pointing to hyperacute T wave and ST-segment depression in lead aVL. B) Repeat ECG obtained after stabilization showed normalization of T wave morphology and normalization of ST-segment depression.

**Image 3:**
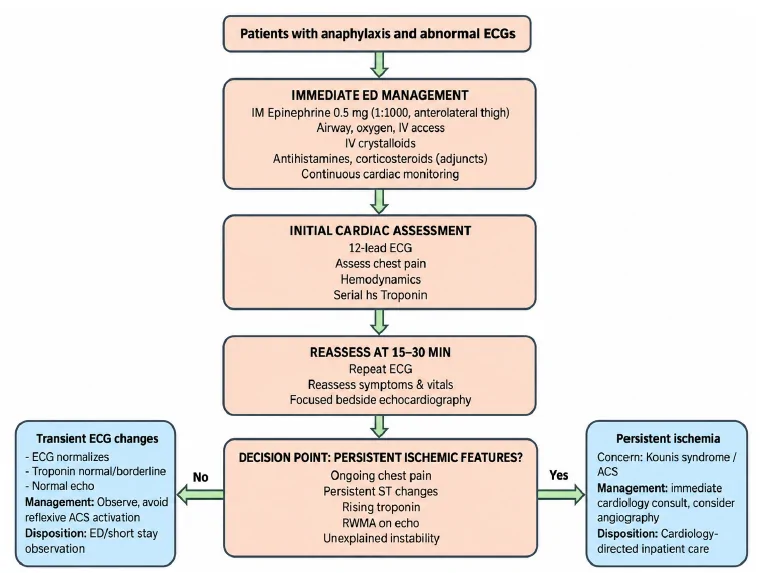
A) The initial 12-lead electrocardiogram demonstrated diffuse ST-segment depression with hyperacute T waves in leads V2–V3. Blue arrow pointing to ST depression with hyperacute T waves. B) A repeat electrocardiogram obtained approximately 30 minutes after clinical stabilization demonstrated complete resolution of hyperacute T waves and ST-segment changes.
